# Exogenous Melatonin Regulates Physiological Responses and Active Ingredient Levels in *Polygonum cuspidatum* under Drought Stress

**DOI:** 10.3390/plants12112141

**Published:** 2023-05-29

**Authors:** Ru-Jie Shi, Ming-Yan Ye, Yue Liu, Qiang-Sheng Wu, Elsayed Fathi Abd_Allah, Nong Zhou

**Affiliations:** 1 College of Food and Biology Engineering, Chongqing Three Gorges University, Chongqing 404120, China; 20050020@sanxiau.edu.cn (R.-J.S.); cqyydye@163.com (M.-Y.Y.); lewyor68@163.com (Y.L.); 2 College of Horticulture and Gardening, Yangtze University, Jingzhou 434025, China; wuqiangsheng@yangtzeu.edu.cn; 3 Plant Production Department, College of Food and Agricultural Sciences, King Saud University, P.O. Box 2460, Riyadh 11451, Saudi Arabia; eabdallah@ksu.edu.sa

**Keywords:** antioxidant enzyme, medicinal plant, melatonin, resveratrol, water stress

## Abstract

*Polygonum cuspidatum*, an important medicinal plant, is rich in resveratrol and polydatin, but it frequently suffers from drought stress in the nursery stage, which inhibits the plant’s growth, active components concentrations, and the price of rhizome in the later stage. The purpose of this study was to analyze how exogenous 100 mM melatonin (MT) (an indole heterocyclic compound) affected biomass production, water potential, gas exchange, antioxidant enzyme activities, active components levels, and *resveratrol synthase* (*RS*) gene expression of *P*. *cuspidatum* seedlings growing under well-watered and drought stress conditions. The 12-week drought treatment negatively affected the shoot and root biomass, leaf water potential, and leaf gas exchange parameters (photosynthetic rate, stomatal conductance, and transpiration rate), whereas the application of exogenous MT significantly increased these variables of stressed and non-stressed seedlings, accompanied by higher increases in the biomass, photosynthetic rate, and stomatal conductance under drought versus well-watered conditions. Drought treatment raised the activities of superoxide dismutase, peroxidase, and catalase in the leaves, while the MT application increased the activities of the three antioxidant enzymes regardless of soil moistures. Drought treatment reduced root chrysophanol, emodin, physcion, and resveratrol levels, while it dramatically promoted root polydatin levels. At the same time, the application of exogenous MT significantly increased the levels of the five active components, regardless of soil moistures, with the exception of no change in the emodin under well-watered conditions. The MT treatment also up-regulated the relative expression of *PcRS* under both soil moistures, along with a significantly positive correlation between the relative expression of *PcRS* and resveratrol levels. In conclusion, exogenous MT can be employed as a biostimulant to enhance plant growth, leaf gas exchange, antioxidant enzyme activities, and active components of *P*. *cuspidatum* under drought stress conditions, which provides a reference for drought-resistant cultivation of *P*. *cuspidatum*.

## 1. Introduction

*Polygonum cuspidatum* Sieb. et Zucc. is a perennial herb that has been used for more than 1500 years as a medicinal plant in China due to its ability to produce a large number of active substances [1]. Among them, its active substances mainly include flavonoids, phenols, and monosaccharides with anti-inflammatory, antibacterial, and antioxidant properties, and thus the extracts of *P*. *cuspidatum* that are mainly composed of resveratrol and emodin exhibit a wide range of antioxidant properties [2]. Resveratrol has been identified as a special secondary metabolite of *P*. *cuspidatum* with anticancer, anti-aging, and antibacterial properties [1]. *P*. *cuspidatum* grows well in humid environments, while it is mostly grown in mountainous areas, where the site conditions are poor and a soil water deficit often occurs, seriously inhibiting its growth and the concentration of active components [1,3,4,5]. In the seedling stage, *P*. *cuspidatum* has poor resistance to the soil water deficit, which negatively affects the plant growth, active components concentrations, and price of later plant rhizomes [3]. Therefore, it is critical to enhance the drought tolerance of *P*. *cuspidatum* seedlings.

Melatonin (MT) is an indole heterocyclic compound with the chemical name N-acetyl-5-methoxytryptamine [6]. MT is present in all major organs of higher plants, such as flowers, leaves, roots, and fruits [7]. The MT in plants can maintain the stability of cell membranes, inhibit chlorophyll decomposition, and increase the antioxidant capacity, which is very beneficial for enhancing the tolerance of plants to abiotic stresses [8]. Therefore, MT has been proposed as a potential regulator of plant growth and development. One of MT’s excellent features was exploited to enhance the stressed resistance of plants. In *Moringa oleifera* plants, exogenous MT with 50, 100, and 150 mM distinctly increased plant growth parameters, antioxidant enzyme activities, and yield components under drought stress (DS), as compared with a non-MT treatment [9]. Rhizospheric application of MT to alfalfa plants accelerated chlorophyll fluorescence and stomatal conductance, along with a greater antioxidant defense system and osmotic adjustment under drought stress [10]. In apple plants, a long-term application of MT dramatically improved N assimilation, including the uptake, transport, and utilization under drought stress [11]. These findings provide support for the involvement of exogenous MT in enhancing the drought tolerance of plants. Exogenous MT application on soybean had important regulatory effects on phenylpropanoid, flavonoid, isoflavonoid, and steroid biosynthesis pathways under drought [12]. Citrus plants treated with MT under drought conditions produced more secondary metabolites such as total flavonoids, total phenols, and essential oil content [13]. However, little work has been carried out around the changes in the active components of medicinal plants under drought stress by exogenous MT.

The aim of this study was to analyze the effects of exogenous MT application on biomass production, gas exchange, water potential, antioxidant enzyme activities, active ingredient levels, and expressions of the *resveratrol synthase* (*RS*) gene in *P*. *cuspidatum* seedlings under well-watered (WW) and DS conditions.

## 2. Results

### 2.1. Response of Plant Biomass

**Figure 1:**
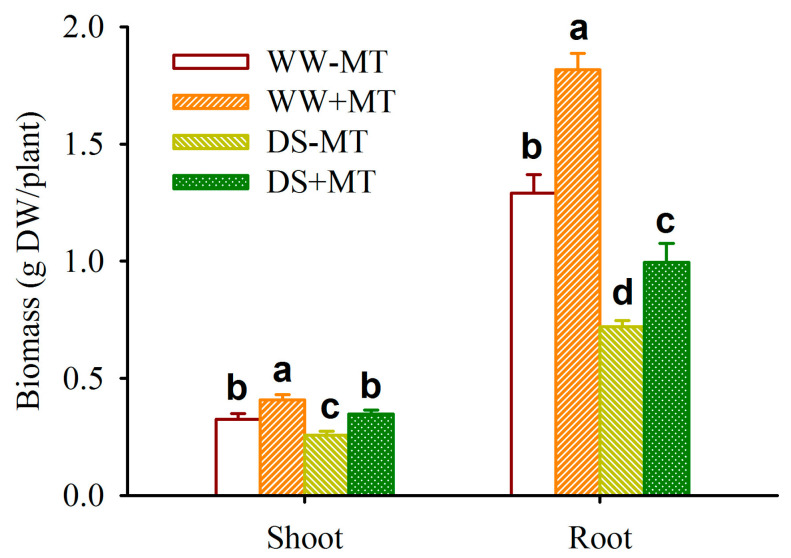
Effects of exogenous melatonin application on shoot and root biomass production of *Polygonum cuspidatum* seedlings exposed to well-watered and drought stress. Data (means ± SD, *n* = 4) followed by different letters above the bars indicate significant (*p* < 0.05) differences. Abbreviations: WW-MT, seedlings treated without exogenous melatonin under well-watered conditions; WW+MT, seedlings treated with exogenous melatonin under well-watered conditions; DS-MT, seedlings treated without exogenous melatonin under drought stress conditions; DS+MT, seedlings treated with exogenous melatonin under drought stress conditions.

Compared with the WW treatment, the DS treatment significantly inhibited the biomass production of *P*. *cuspidatum* by 20.77% and 14.72% in the shoot biomass of non-MT- and MT-treated plants, respectively, and by 44.19% and 45.25% in the root biomass of non-MT- and MT-treated plants, respectively (Figure 1). However, exogenous MT application significantly increased shoot and root biomass production by 26.38% and 34.95% under WW conditions and 40.29% and 38.19% under DS conditions, respectively.

### 2.2. Response of Leaf Gas Exchange and Water Potential

**Figure 2:**
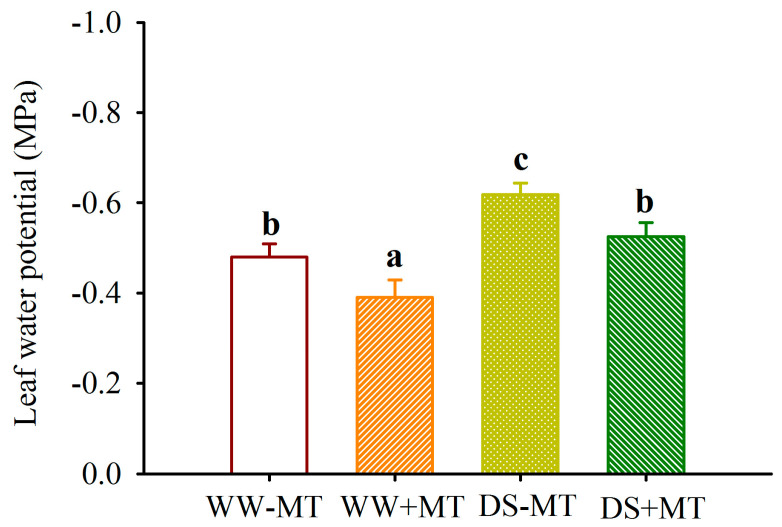
Effects of exogenous melatonin application on leaf water potential of *Polygonum cuspidatum* seedlings exposed to well-watered and drought stress. Data (means ± SD, *n* = 4) followed by different letters above the bars indicate significant (*p* < 0.05) differences. See Figure 1 for the abbreviation.

**Figure 3:**
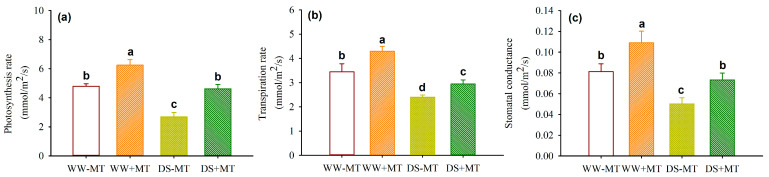
Effects of exogenous melatonin application on leaf photosynthetic rate (**a**), transpiration rate (**b**), and stomatal conductance (**c**) of *Polygonum cuspidatum* seedlings exposed to well-watered and drought stress. Data (means ± SD, *n* = 4) followed by different letters above the bars indicate significant (*p* < 0.05) differences. See Figure 1 for the abbreviation.

The drought treatment significantly decreased the leaf water potential by 28.65% in MT-untreated plants and 34.62% in MT-treated plants, respectively, compared with the WW treatment (Figure 2). Furthermore, the DS treatment also triggered a decrease in the leaf photosynthetic rate, transpiration rate, and stomatal conductance by 43.83%, 30.48%, and 38.15% in MT-untreated plants, respectively, and by 25.99%, 31.39%, and 32.80% in MT-treated plants (Figure 3a–c). However, exogenous MT treatment significantly increased leaf water potential by 18.75% and 14.98% under WW and DS conditions, respectively. Similarly, MT-treated plants had a significantly higher leaf photosynthetic rate, transpiration rate, and stomatal conductance by 30.46%, 24.60%, and 34.15% under WW, and 71.91%, 22.94%, and 45.77% under DS.

### 2.3. Response of Root Antioxidant Enzyme Activities

**Figure 4:**
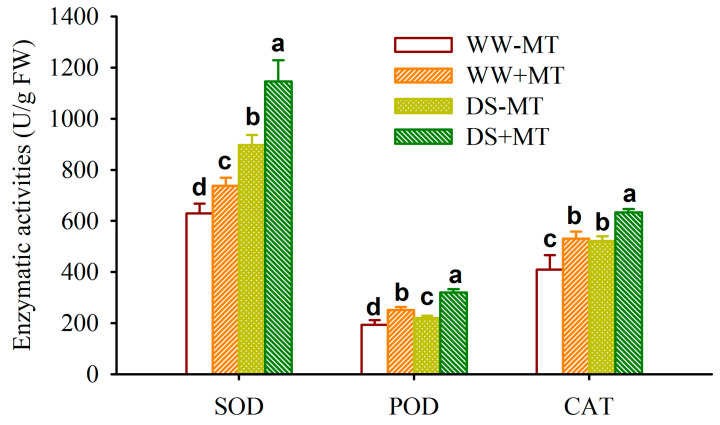
Effects of exogenous melatonin application on leaf superoxide dismutase (SOD), peroxidase (POD), and catalase (CAT) activities of *Polygonum cuspidatum* seedlings exposed to well-watered and drought stress. Data (means ± SD, *n* = 4) followed by different letters above the bars indicate significant (*p* < 0.05) differences. See Figure 1 for the abbreviation.

Compared to WW treatment, DS treatment significantly increased root superoxide dismutase (SOD), peroxidase (POD), and catalase (CAT) activities by 42.44%, 13.30%, and 27.08% in MT-untreated plants and 55.43%, 26.77%, and 19.47% in MT-treated plants, respectively (Figure 4). Exogenous MT application, on the other hand, significantly increased root SOD, POD, and CAT activities by 17.05%, 30.04%, and 29.41% under WW and by 27.73%, 45.50%, and 21.66% under DS, respectively.

### 2.4. Response of Root Active Substance Concentrations

**Figure 5:**
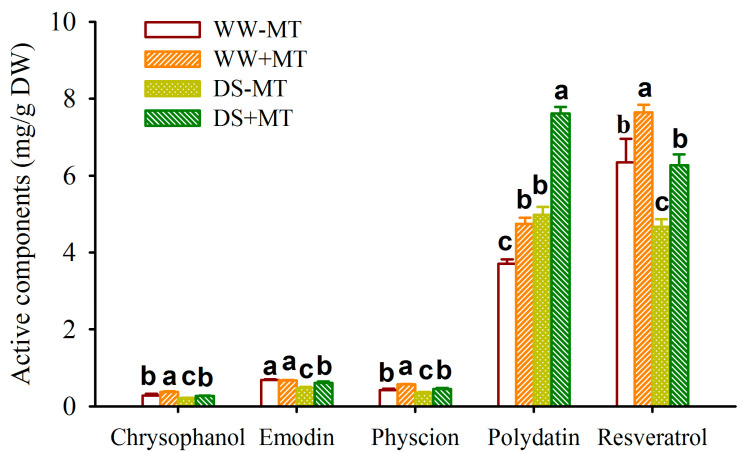
Effects of exogenous melatonin application on root chrysophanol, emodin, physcion, polydatin, and resveratrol concentrations of *Polygonum cuspidatum* seedlings exposed to well-watered and drought stress. Data (means ± SD, *n* = 4) followed by different letters above the bars indicate significant (*p* < 0.05) differences. See Figure 1 for the abbreviation.

DS treatment significantly promoted root polydatin levels by 34.39% and 60.48% in non-MT-treated and MT-treated plants, respectively, compared to WW treatment (Figure 5). However, for other active ingredients, including chrysophanol, emodin, physcion, and resveratrol, DS treatment significantly inhibited their levels by 22.76%, 31.10%, 14.30%, and 26.38% respectively in non-MT treated plants, and by 26.57%, 9.70%, 20.90%, and 18.02% respectively, in MT-treated plants (Figure 5). On the other hand, MT did not change the root emodin levels under WW conditions, but it significantly increased root chrysophanol, physcion, polydatin, and resveratrol levels by 31.82%, 36.56%, 27.98%, and 20.58%, respectively, compared to the non-MT treatment. Under DS conditions, MT treatments significantly increased root chrysophanol, emodin, physcion, polydatin, and resveratrol levels by 25.32%, 28.57%, 26.05%, 52.83%, and 34.28%, respectively.

### 2.5. Response of Root PcRS Gene Expression

**Figure 6:**
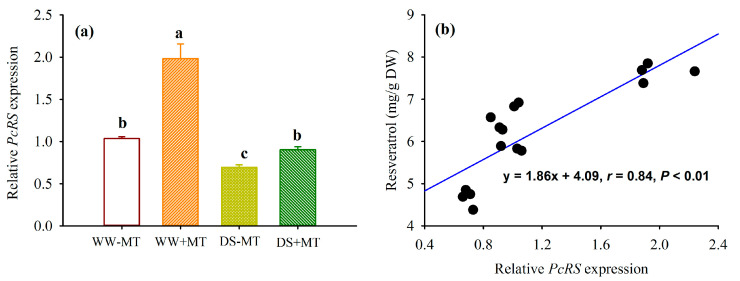
Expressions of *resveratrol synthase* (*PcRS*) gene (**a**) and its correlation with resveratrol levels (**b**) in roots of *Polygonum cuspidatum* seedlings exposed to well-watered and drought stress and applied by exogenous melatonin. Data (means ± SD, *n* = 4) followed by different letters above the bars indicate significant (*p* < 0.05) differences. See Figure 1 for the abbreviation.

DS treatment significantly down-regulated the *resveratrol synthase* (*PcRS*) gene expression in non-MT- and MT-treated plants by 0.33- and 0.54-fold, respectively, compared to the WW treatment (Figure 6a). However, the application of exogenous MT significantly up-regulated the *PcRS* gene expression by 0.92- and 0.30-fold under WW and DS conditions, respectively, compared to the non-MT application. In addition, the relative expression of the root *PcRS* was significantly (*p* < 0.01) and positively correlated with root resveratrol concentrations (Figure 6b).

## 3. Discussion

The results of this study revealed that although DS treatment greatly inhibited the biomass production of *P*. *cuspidatum* seedlings, exogenous MT treatment could dramatically ameliorate the inhibitory effect, with a greater improvement under DS than under WW. This is consistent with the results obtained by Altaf et al. [14] in applying MT to tomatoes under drought conditions. Changes in the shoot and root biomass showed that 100 mM MT application had the desired effect on *P*. *cuspidatum* seedlings under drought conditions. MT can promote cell expansion and participate in various endogenous hormone signaling pathways, such as auxins, thus synergistically regulating plant growth and development [15]. However, it is unclear whether endogenous MT also functions as described above in *P*. *cuspidatum* and needs to be investigated in depth.

The present study also showed that drought treatment significantly inhibited leaf gas exchange, regardless of whether exogenous MT was used. This implies that the soil water deficit in this study reduced stomatal conductance and CO_2_ diffusion into the leaf, thus causing the inhibition of photosynthesis [16]. However, exogenous MT treatment significantly increased the leaf photosynthetic rate, transpiration rate, and stomatal conductance, under both WW and DS conditions. This suggests that MT is able to mitigate the restriction of leaf gas exchange caused by drought. This result is consistent with the application of MT to the drought-sensitive variety (Qinyou 8) of *Brassica napus* plants [17]. Such improved results are attributed to the fact that MT protects plants from drought-induced photoinhibition and photooxidation, particularly via sustaining photosystem II activity [18].

This study showed that drought treatment increased the activities of SOD, POD, and CAT in roots of *P*. *cuspidatum*, indicating that the antioxidant enzyme defense system of *P*. *cuspidatum* was activated by drought. Therefore, *P*. *cuspidatum* still had a certain potential for drought tolerance. Hu et al. [19] also found that the up-regulation of the *PcDREB2A* gene encoding a DRE-binding transcription factor enhanced the drought tolerance of *P*. *cuspidatum*. The activities of SOD, POD, and CAT were further increased when *P*. *cuspidatum* was treated with exogenous MT, indicating that MT-treated plants maintained higher antioxidant enzyme activities than non-MT-treated plants, which was crucial for them to scavenge more reactive oxygen species and maintain low oxidative damage under drought. As an electron donor, MT can directly react with reactive oxygen species in plants and also acts as an antioxidant with a stronger peroxyl radical scavenging activity than vitamin E, allowing the body to maintain low levels of reactive oxygen species [20].

Earlier studies have shown that active substances of *P*. *cuspidatum* can be modulated by external stimuli such as inoculation with *Funneliformis mosseae* and *Piriformospora indica* singly or in combination, as well as an additional supply of phosphorus fertilizer [4,21]. In this study, the soil water deficit dramatically reduced levels of most root active compounds, including chrysophanol, emodin, physcion, and resveratrol, with the exception of increased polydatin. Similarly, Xie et al. [22] discovered in *Glycyrrhiza uralensis* plants that moderate drought treatment significantly promoted root glycyrrhizin levels, while inhibiting root liquiritin levels. This suggests that the active ingredient levels of *P*. *cuspidatum* are variable under drought conditions. Jayalakshmi and Devika [23] also reported that polydatin derived from *P*. *cuspidatum* presented a strong antioxidant activity in vitro.

In this study, exogenous MT application significantly raised the levels of the five active components studied in the roots of *P*. *cuspidatum* under both WW and DS conditions, with the exception of unchanged emodin under WW conditions. Moreover, MT-triggered increases in polydatin and resveratrol levels of *P*. *cuspidatum* were greater under DS conditions than under WW conditions. This suggests that exogenous MT has the ability to increase the amounts of active components of *P*. *cuspidatum* and may also be involved in the synthesis of active components of *P*. *cuspidatum*. It is known that MT is an endogenous signal of darkness that can represent physiological effects in plants by interacting with plasma membrane MT receptors as well as calmodulin [24]. In addition, MT may also have some auxin-like effects on plant growth [25], thus accelerating plant growth responses as well as the accumulation process of active ingredients. Therefore, the mechanism of how MT affects the active components of *P*. *cuspidatum* needs to be further investigated.

Resveratrol is synthesized by the catalysis of RS with one molecule of coumaroyl-CoA and three molecules of malony-CoA [26]. Transgenic plants with *RS* overexpression produced more resveratrol [27]. In this work, DS dramatically suppressed the expression of root *RS*, irrespective of whether MT was used, along with a reduction in root resveratrol. As a consequence, there was a significantly (*p* < 0.01) positive correlation between the root *RS* expressions and root resveratrol. Sun et al. [5,21] also found the up-regulation of *PcRS* expressions by inoculation with *Funneliformis mosseae* as well as a P supply. This implied that MT treatment promotes resveratrol synthesis by up-regulating the expression level of *RS*. However, besides RS, stilbene synthase and resveratrol-forming stilbene synthase are involved in the biosynthesis of resveratrol [26]. Expressions of these enzyme genes can also be affected by the external environment [5,21], and additional studies are needed to reveal whether exogenous MT also regulates the expression levels of other genes.

## 4. Conclusions

The application of exogenous MT significantly improved the biomass and promoted the leaf gas exchange, leaf water potential, antioxidant enzyme activities and active compositions concentrations under both WW and DS conditions, along with the up-regulation of *PcRS*. The increase in polydatin and resveratrol in *P*. *cuspidatum* triggered by exogenous MT was more pronounced under drought conditions than under WW conditions. An MT-induced increase in resveratrol was associated with an up-regulated *RS* expression induced by MT. Therefore, MT can be properly introduced as a biostimulant in *P*. *cuspidatum* at the nursery stage. Nevertheless, the present study only examined the effects of exogenous MT. More studies utilizing MT inhibitors are required to determine whether and how endogenous MT is directly involved in physiological responses. In addition, molecular mechanisms of how MT accelerates the increase in active components of *P*. *cuspidatum* remain to be further studied.

## 5. Materials and Methods

### 5.1. Plant Culture and Treatment Application

The seed of *P*. *cuspidatum* cv. Fangxianhuzhang from the Shiyan Academy of Agricultural Sciences was germinated in a bed containing vermiculite, sand, and soil in a volume ratio of 1:1:3, without the use of any environmental control equipment. After about 40 days, identical seedlings with three leaves were transplanted into a 2.6-L plastic pot with the substrate of vermiculite and soil in the volume ratio of 1:2. The transplanted seedlings were placed in a greenhouse of 29 °C/23 °C (day/night temperature, 16 h/8 h), with a photo density of 910 μmol/m^2^/s and relative humidity of 65%. After this, the plants were watered every three days. After five weeks of acclimation, these seedlings were treated with exogenous MT as well as drought treatments.

The soil water regimes used were 100% field capacity (WW) and 75% field capacity (DS), respectively. The pots were weighed daily to control the intensity of the water treatments. Based on the results of Sadak et al. [9] and our previous preliminary experiment, we chose 100 mM MT as the treatment concentration, in which 0.1% Tween-20 was mixed as a surfactant. Exogenous MT application was arranged with 30 mL of 0 and 100 mM MT/pot, and the frequency was carried out at three-day intervals, for a total of six times.

### 5.2. Experimental Design

This experiment was arranged in four treatments: (i) *P*. *cuspidatum* seedlings applied with 100 mM MT under WW conditions (WW+MT), (ii) *P*. *cuspidatum* seedlings applied with 0 mM MT under WW conditions (WW-MT), (iii) *P*. *cuspidatum* seedlings applied with 100 mM MT under DS conditions (DS+MT), and (iv) *P*. *cuspidatum* seedlings applied with 0 mM MT under DS conditions (DS-MT). Each treatment had 8 replicates with a total of 32 pots in a completely randomized block arrangement.

### 5.3. Determinations of Plant Biomass, Leaf Gas Exchange, and Leaf Water Potential

After 12 weeks of DS treatment, the plants were harvested and their biomass was weighed. Four potted plants from each treatment were immediately frozen in liquid nitrogen and preserved at −80 °C for gene expression analysis, while the other plants were dried at 75 °C until reaching constant weight.

On a sunny day before harvest, the leaf gas exchange variables including photosynthetic rate, transpiration rate, and stomatal conductance were monitored by a portable photosynthesis analyzer (Li-6400, Li-COR Inc., Lincoln, NE, USA). On the day of the harvest, the leaf water potential was measured using a portable plant water potential pressure chamber (1515D; PMS Instruments Co., Albany, OR, USA) starting at 9:30 a.m., according to the user manual.

### 5.4. Determinations of Antioxidant Enzyme Activities in Leaves

A 0.25 g leaf sample was ground with 6 mL of 0.1 mol/L phosphate buffers (pH 7.8) and then centrifuged at 3000× *g*/min for 15 min. The supernatant was collected for the purpose of analyzing the antioxidant enzyme activities. The SOD activity was determined by the inhibition of nitroblue tetrazolium (NBT) reduction [28], where the reaction solution consisted of 0.1 mol/L phosphate buffer, 0.75 mmol/L NBT, 130 mmol/L methionine solution, 0.1 mmol/L EDTA-Na_2_, 0.02 mmol/L riboflavin, and the supernatants. The POD activity was determined by the guaiacol method [29], with the reaction solution consisting of 0.1 mol/L phosphate buffer, 2% hydrogen peroxide, 0.05 mmol/L guaiacol agro, and 0.1 mL the supernatants. The CAT activity was determined by the colorimetric method [30], where 0.1 M hydrogen peroxide was added to the enzyme solution for reaction, and the change in the absorbance value at 240 nm was measured within 1 min.

### 5.5. Determinations of Active Components in Roots

Root active components (chrysophanol, emodin, physcion, polydatin, and resveratrol) were extracted by the method described by Sun et al. [21]. Briefly, sieved (2 mm) dried root samples were incubated with 80% methanol solutions for 30 min under sonication conditions and centrifuged at 4000× *g*/min for 10 min. The supernatant was collected and passed through 0.22 μm filter membranes for HPLC assay. HPLC conditions were carried out by the protocol as described by Sun et al. [21], where the HPLC was LC-20AT (Shimadzu, Tokyo, Japan), and the mobile phases were acetonitrile (phase A) and 0.1% formic acid (phase B). In addition, standards of polydatin (CAS: 27208-80-6), resveratrol (CAS:501-36-0), emodin (CAS:518-82-1), physcion (CAS:521-61-9), and chrysophanol (CAS:481-74-3) were purchased from Chengdu Push Bio-technology Co., Ltd. (Chengdu, China) and dissolved in methanol solutions before assaying.

### 5.6. Determinations of Gene Expressions in Roots

The total RNA of the roots was extracted using the Quick RNA Isolation Kit and then further reversely transcribed into cDNA using the TRUE 1st Strand cDNA Synthesis Kit with a gDNA Eraser. Based on the results of Sun et al. [5], the *PcRS* gene (ID: DQ900615.1) was chosen, and their specific sequences were designed by the Primer Premier 5.0, with the forward sequence being GAGATGACGAAGGCACTAACA (5′→3′) and the reverse sequence being GGAAGTAGAAGTCGGGAAAGTC (5′→3′). qRT-PCR was performed in the CFX96 real-time PCR detection system with β-actin as the housekeeping gene. Three biological replicates were available for each treatment. The relative expression of genes was calculated using the 2^−ΔΔCt^ method [31], and the results were standardized to the WW-MT treatment.

### 5.7. Statistical Analysis

One-way analysis of variance (ANOVA) was used to analyze the data collected from the experiment. Significant (*p* < 0.05) differences between treatments were analyzed using Duncan’s multiple range tests. These analyses were performed with SAS software v9.4 (SAS Inc., Cary, NC, USA).

## Data Availability

All the data supporting the findings of this study are included in this article.
